# The use of ultrasound in clinical setting for children affected by NAFLD: is it safe and accurate?

**DOI:** 10.1186/1824-7288-37-36

**Published:** 2011-08-02

**Authors:** Valerio Nobili, Claudia Della Corte, Lidia Monti, Anna Alisi, Ariel Feldstein

**Affiliations:** 1 Valerio Nobili, Unit of Liver Research of Bambino Gesù Children's Hospital, IRCCS, P.le S. Onofrio 4; 00165 Rome, Italy; 2 Claudia Della Corte, Unit of Liver Research of Bambino Gesù Children's Hospital, IRCCS, P.le S. Onofrio 4; 00165 Rome, Italy; 3 Lidia Monti, Radiology Department of Bambino Gesù Children's Hospital, IRCCS, P.le S. Onofrio 4; 00165, Rome, Italy; 4 Anna Alisi, Unit of Liver Research of Bambino Gesù Children's Hospital, IRCCS, P.le S. Onofrio 4; 00165 Rome, Italy; 5 Ariel Feldstein, Department of Pediatric Gastroenterology, Cleveland Clinic, 9500 Euclid Avenue, Cleveland, OH 44195, USA

## Abstract

Non-alcoholic fatty liver disease (NAFLD) has become over the last decade the most common form of chronic liver disease in children and adults. Thus, establishing the diagnosis of NAFLD is of utmost importance and represents a major challenge as the disease is generally silent and the current gold standard for diagnosis is an invasive liver biopsy, a procedure that is not suitable for screening purposes. Many non-invasive diagnostic tools have been evaluated so far. Recently the utility of ultrasonography for non-invasive diagnosis and estimation of hepatic steatosis has been demonstrated in a large prospective pediatric study.

## 

Non-alcoholic fatty liver disease (NAFLD) has become over the last decade the most common form of chronic liver disease in children and adults. It is tightly associated with obesity and threatens to become a serious public health problem in the United States and many other countries. NAFLD is estimated to affect close to 10% of the American population aged 2 to 19 years, and this figure increases to 30 - 40% among obese children.

In addition to being a highly common condition in children, several lines of evidence suggest that NAFLD is a potentially serious condition. A recent long-term longitudinal study has demonstrated that similarly to adults, NAFLD in children is a disease with the potential to progress [1]. Some children in this study presented with cirrhosis at time of diagnosis, others showed progressive liver disease resulting in significant liver-related morbidity. Moreover, the presence of NAFLD in children may be a key indicator of the metabolic status and a good predictor for development of type 2 diabetes. Thus, establishing the diagnosis of NAFLD in children is of utmost importance and represents a major challenge as the disease is generally silent and the current gold standard for diagnosis is an invasive liver biopsy, a procedure that is not suitable for screening purposes.

Other screening measures have been employed, including monitoring liver transaminases and, recently, the American Academy of Paediatrics has recommended that serum aminotransferases (ALT and AST) should be performed in all overweight children starting at age 10 years if their BMI is ≥ 95th percentile or between 85-94th percentile with risk factors. ALT and AST are to be checked in addition to fasting glucose and lipid profile. However, it has become clear that in both adults and children liver enzymes perform poorly for NAFLD diagnosis with two-thirds of NAFLD patients showing normal levels of serum ALT and AST [2].

Regarding the use of imaging for screening purposes, hepatic ultrasonography (US) is the most commonly used modality largely because it is relatively inexpensive, widely available and is user-friendly [3]. Several studies in adults have demonstrated that this technique is highly sensitive and specific for detection of NAFLD. Moreover, hepatic US can provide a good estimate of the degree or extent of hepatic steatosis present based on a series of US characteristics including hepatorenal echo contrast, liver echogenicity, visualization of intrahepatic vessels, and visualization of liver parenchyma and the diaphragm [4-7]. In the recent years a computer-assisted method for measuring liver echogenicity calculating the hepatorenal index has been evaluated with good results [8]. However, the diagnostic accuracy of hepatic US and the utility for quantification of the degree of hepatic steatosis in children remain unknown.

Recently Dr Shannon A, from the Department of Paediatric Gastroenterology, Cleveland Clinic, Cleveland, Ohio, headed by Prof Feldstein A, conducted a study to evaluate the utility of hepatic US for quantifying hepatic steatosis in a large well-characterized paediatric population with biopsy-proven NAFLD.

The manuscript entitled "Ultrasonographic Quantitative Estimation of Hepatic Steatosis in Children with Nonalcoholic Fatty Liver Disease (NAFLD)" will appear on JPGN-NA in the August issue, and it will represent the largest cohort of children with biopsy proven NAFLD reported up to date [9]. In this study the Authors demonstrate the utility of hepatic ultrasonography for non-invasive diagnosis and estimation of hepatic steatosis in children, showing a tight correlation between ultrasonographic steatosis score (USS) and severity of steatosis on liver biopsy [9]. This study confirms the US as now the best imaging technique for the assessment of liver steatosis, because it is a safe, non-invasive and non-expensive diagnostic tool. In fact, in the recent years, magnetic resonance imaging (MRI) has also been shown to be able to demonstrate and quantify fat infiltration of the liver [10,11]. However, it has been proved of limited application for the study of children because of its lengthy scan times, its reliance on compliance of the patients, with usually necessity of conscious sedation, and its high costs. Whereas, the user-friendliness of the US makes it a useful test for screening programs in paediatric patients with suspected NAFLD.

The strong correlation between US and histological parameters of fatty liver indicates that hepatic US can be used in the monitoring of steatosis after therapeutic interventions in children. Therefore clinical trials having steatosis as end-point can correctly use serial hepatic US to estimate the response to treatment. Recently Nobili V and co-workers demonstrated the improvement of liver steatosis in children with NAFLD following docosahexaenoic acid (DHA) supplementation [12]. In this randomized placebo-controlled trial liver biopsy and hepatic US were performed at baseline. After 6 months of treatment the improvement in hepatic steatosis was evaluated by US examination.

In conclusion this paper disclose the way for a rationale use of US for non-invasive diagnosis of fatty liver and for estimation of steatotis with particular regard to monitoring therapeutic efficacy, leaving the liver biopsy only for definition of inflammatory activity and fibrosis stage.
